# Pre-adult onset and patterns of suicidality in patients with a history of recurrent depression

**DOI:** 10.1016/j.jad.2011.12.011

**Published:** 2012-04

**Authors:** J. Mark G. Williams, Thorsten Barnhofer, Catherine Crane, Danielle S. Duggan, Dhruvi Shah, Kate Brennan, Adele Krusche, Rebecca Crane, Catrin Eames, Mariel Jones, Sholto Radford, Ian T. Russell

**Affiliations:** a University of Oxford, UK; b Bangor University, UK

**Keywords:** Keywords, Depression, Suicidality, Suicide attempts, Early onset, Pre-adult onset

## Abstract

**Background:**

This report assesses the association between age of onset of major depression and later suicidality in a sample of 276 recurrently depressed patients recruited for the Oxford/Bangor Staying Well after Depression (SWAD) Trial, and interviewed when in remission.

**Methods:**

The study enrolled adult patients with a history of at least three episodes of non-psychotic major depressive disorder from primary care and psychiatric care practices and through community advertisements. At study entry, all participants estimated the age of their first onset of a major depressive episode and completed both self-report and interview-based assessments of past and current suicidal ideation and behavior. Participants were divided into pre-adult and adult onset groups using a cut-off age of 18.

**Results:**

Forty-eight percent of the sample reported a pre-adult age of onset. Pre-adult age of onset was significantly associated with suicidality, both from self-report and from interviewer assessment even when adjusting for differences in age, gender, employment status, length of the disorder and early adversity.

**Limitations:**

Relevant variables were all assessed through retrospective reports.

**Conclusions:**

Pre-adult age of onset is closely associated with risk for and severity of later suicidality, replicating, in a sample of patients assessed when in remission, findings from studies that assessed patients when currently depressed. The association of pre-adult age of onset with suicidality is not due to differences in sociodemographic variables, length of the disorder and early adversity.

## 1 Introduction

Depression remains the largest contributor to Years of Life Lost to Disability across middle and high income countries, and is third even for low income countries (Eaton et al., 2008). Understanding the etiological factors that predict the most serious patterns of depression across the lifespan is therefore a priority. The age of onset of depression has long been recognized as a consistent long-term predictor of morbidity. In particular, if the first episode of depression occurs before 18 years of age, depression in later life is more likely, more persistent and more recurrent (Coryell et al., 2009; Klein et al., 1999; Kupfer et al., 1989; Pine et al., 1998; Weissman et al., 1984; Weissman et al., 1988; Zisook et al., 2007).

Coryell et al. (2009) reported data from a twenty-year follow-up of patients (*N* = 220) from the NIMH Collaborative Program of Psychobiology of Depression and found not only that age of onset affected morbidity (as measured by the proportion of time spent in episode), but that this effect occurred independently of current age at presentation for treatment. Importantly, the pattern of morbidity, once determined by age of onset, did not then change over the twenty year follow-up period for any age range studied (from 20 s to 40 s; from 30 s to 50 s, or from 50 s to 70 s). These authors conclude: “The tendency of an individual's depressive illness to persist or recur appears to declare itself early and to be an enduring quality” (Coryell et al., 2009, p. 1693).

This pattern of data would not be so significant if there were relatively few people who first become depressed when young. However, data from the Epidemiological Catchment Area study found that during the second half of the twentieth century, there were pronounced secular trends towards increasingly younger age of onset of major depression (Burke et al., 1991). This pattern from epidemiological research has been largely confirmed by later studies. Zisook et al. (2007) reported a mean age of onset of major depression in patients recruited to the STAR*D of 26 years, with 37% of the sample reporting a pre-adult age of onset and the modal age of onset being between 13 and 15 years of age. Similar data have emerged from other clinical studies (Alpert et al., 1999; Fava et al., 1996), although some recent research suggests that the percentage of patients with pre-adult onset may be somewhat lower in more naturalistic patient samples that have not been recruited for the purpose of clinical trials (Van Noorden et al., 2011). In addition to a more recurrent and persistent course of the disorder, pre-adult age of onset has been found to be associated with a number of other indicators of severity of psychopathology, including higher familiality of depression (Kendler et al., 2005; Klein et al., 1999; Parker et al., 2003; Zisook et al., 2007), higher levels of childhood adversity (Jaffee et al., 2002), higher psychiatric comorbidity, particularly with anxiety disorders and alcohol or drug abuse (Klein et al., 1999; Parker et al., 2003; Van Noorden et al., 2011; Zisook et al., 2007) and, most importantly and concerningly, increased suicidality (Van Noorden et al., 2011; Zisook et al. 2004, 2007). Patients with pre-adult onset of the disorder not only show higher rates of suicide attempts but also report higher degrees of suicidal intent when they become suicidal (Thompson, 2008).

However, authors of previous studies have pointed out a number of limitations. Among them is the fact that in all of the previous studies patients were assessed when depressed, leading to possible biases in estimates of age of onset and other aspects of self-reported symptoms of depression. Additionally, the nature of the studies necessitated recruiting across the whole range of severity of patients seeking treatment for major depression, so the question arises whether the same pattern would be observed if only a more chronic or recurrent group of patients were studied. This is particularly important in light of findings suggesting that it is not pre-adult onset of depression per se that is problematic but more specifically pre-adult onset followed by a recurrent or chronic course (Hammen et al., 2008). The current study investigated the same variables investigated in previous research in a sample of patients with recurrent major depression. Because the patients had been recruited for a trial of a *preventative* treatment (the Staying Well after Depression study of Mindfulness-based Cognitive Therapy) the study protocol required that all patients were currently in remission and had been so for a minimum of two months.

The study was therefore able to address a number of hypotheses. First, we predicted that this sample with a history of at least three episodes of non-psychotic major depressive disorder would show the same pattern of age of onset as reported from previous research, even though patients were currently in remission. Second, that pre-adult onset of the disorder would be closely associated with increased suicidality, assessed both through self-report and interview-based measures of suicide attempts and ideation. We also compared participants with pre-adult and adult onset of the disorder with regard to other indicators of psychopathology including indicators of the course of the depression, current symptoms and current and past diagnoses of other psychiatric disorders as well as childhood adversity. Furthermore we wished to examine the data to see if any association between age of onset and suicidality could be accounted for by differences in sociodemographic variables, such as age, gender, and employment status. Finally, as previous studies had used different cut-offs to define pre-adult versus adult onset, we repeated analyses for a different cut-off age in order to test generalizability.

## 2 Method

### 2.1 Participants and procedure

The rationale and design of the SWAD trial have been described in detail elsewhere (Williams et al., 2010). Briefly, the Staying Well after Depression (SWAD) Trial aims to test the effectiveness of a form of psychological treatment, Mindfulness-Based Cognitive Therapy (MBCT), in preventing relapse to suicidal depression against an active control treatment and treatment as usual. Participants of this study were the intake sample of the SWAD Trial. Inclusion and exclusion criteria for the trial ensured that patients entering the study had a history of highly recurrent depression stratified for level of suicidality prior to randomization, but were in recovery or remission at the time of entry. In particular participants were included based on the following criteria: a) age between 18 and 70 years, b) meeting enhanced DSM-IV-TR (American Psychiatric Association, 2000) criteria for a history of Recurrent Major Depression, namely a history of at least three episodes of depression, of which two must have occurred within the last five years, and one within the last two years, c) meeting NIMH guidelines for recovery or remission at the time of baseline assessment, i.e. no more than one core symptom or suicidality and one other symptom over a period of at least one week during the previous eight weeks, d) giving informed consent, and e) consent from the participant's General Practitioner (GP). Potential trial participants were excluded if one or more of the following criteria applied: a) history of schizophrenia, schizoaffective disorder, bipolar I disorder, current severe substance abuse, organic mental disorder, pervasive developmental delay, a primary diagnosis of obsessive–compulsive disorder or eating disorder, or regular self-harm, b) a positive continuing response to CBT, c) currently receiving psychotherapy or counseling more than once a month, and d) not able to complete the baseline research assessment, for example because of difficulties with English, visual impairment, or cognitive difficulties. Participants were recruited through advertisements in the community, in clinics and GP surgeries, as well as through referrals from GPs and mental health clinicians. All risks, benefits, and adverse events were explained to the participants, who gave their written informed consent before the assessment of eligibility. The study had received ethical approval from the Oxfordshire Research Ethics Service C and the North Wales Research Ethics Committee. After an initial telephone screening participants came to the research facilities where a structured interview was conducted and participants filled in self-report questionnaires.

### 2.2 Assessment and definition of age of onset

The age of onset of each participant's first episode of Major Depression was recorded as part of the Structured Clinical Interview for DSM-IV (SCID-IV, First et al., 2002) in which the participant's history of depression and presence or absence of other mental disorders was assessed. The SCID module for Major Depression assesses fulfillment of criteria for Major Depression according to DSM-IV for potential present and past episodes of depression. In the current context present symptoms of depression were assessed in order to ascertain that participants were in recovery or remission. Interviewers then checked criteria for an episode that participants described as their worst ever and following that assessed other potential past episodes in reverse chronological order. Age of onset was determined by asking participants to indicate their age at the time of their first episode that met criteria for Major Depression. If the participant reported a gradual onset of symptoms in which the first Major Depressive Episode was superimposed on pre-existing dysthymic disorder, they were asked to give a best estimate of when depression first reached the level of a full episode over a period of at least two weeks.

Previous research has used cut-off points to differentiate pre-adult onset and adult onset ranging from 15 (Hammen et al., 2008), 17 (Jaffee et al., 2002), 18 (Alpert et al., 1999; Benazzi, 2000; Fava et al., 1996; Zisook et al., 2007), 21 (Klein et al., 1999), and 25 (Parker et al., 2003) to 30 years (Thompson, 2008) with most of the studies using a cut off of 18. In order to maintain comparability with most of the currently published research we therefore defined pre-adult onset as onset at an age < 18 and adult onset as onset at an age ≥ 18. Additionally, as a means to see whether our findings would generalize over a broader range of cut-off ages we conducted sensitivity analyses by re-analyzing data with a cut-off age of 21 following the example of van Noorden et al. (2011).

### 2.3 Assessment and definition of demographic and dependent variables

Demographic variables were derived from information given at the start of the structured interview. They included current age, gender, employment status, which for the current study was broken down into the categories employed, unemployed, and retired, and ethnicity, for the current purpose broken down into Caucasian-white and other.

Information regarding past suicidality was derived from a number of different self-report and interview-based sources. Self-reported information regarding the presence of past suicide attempts was derived from the Beck Scale for Suicidal Ideation (BSS, Beck and Steer, 1993). For the current purpose a binary measure of presence versus absence of past suicide attempts was derived from item 20 of this questionnaire, which explicitly asks participants whether they have never, once, or twice or more attempted suicide. As an interview-based measure of past suicidality we used the Scale for Assessment of Suicide and Self-Injury (SASII, Linehan et al., 2006) to ascertain presence versus absence of previous episodes of suicidality and self-harm. Furthermore, we assessed diagnostic information about suicidality during the worst past episode of depression as part of the Structured Clinical Interview for DSM-IV (SCID-IV). Participants were asked to report on suicidality during their worst episode of depression and interviewers rated severity on a scale ranging from 0 indicating no suicidality to 5 indicating an actual suicide attempt.

The SCID-IV, as all other interview-based assessments, was conducted by trained research psychologists. The structured interview yielded information on current and past depression and other psychiatric disorders as well as current and past treatments. The main variables derived from the interview to characterize the course of the depression were length of the disorder, computed as current age minus age of onset, the number of episodes of depression and presence versus absence of an episode of chronic depression as defined by DSM-IV, i.e. an episode of Major Depression that lasted for at least two years. In order to compare groups with regard to whether they were currently suffering from an anxiety disorder or had suffered from an anxiety disorder in the past, we coded presence versus absence of anxiety disorders based on current and past diagnoses in the SCID anxiety disorders section. It is important to note in this context that exclusion criteria for the trial included the diagnosis of an obsessive–compulsive disorder. In a similar way we derived a binary measure of past history of substance dependence or abuse from SCID diagnoses of past alcohol- and substance dependence or abuse. Current use of antidepressants was assessed by asking participants to list any antidepressant medication they had taken during the last 7 days.

Current level of depressive symptoms was assessed using the Hamilton Rating Scale for Depression (HAMD, Hamilton, 1960) as well as the Beck Depression Inventory-II (BDI-II, Beck et al., 1996). Internal consistency for these measures in our study was Cronbach's α = .73 and .90, respectively. In addition current hopelessness was assessed using the Beck Hopelessness Scale (BHS, Beck, 1988), Cronbach's α = .89. Early adversity was assessed through self-reports on the Childhood Trauma Questionnaire (CTQ, Bernstein and Fink, 1998). The CTQ consists of 28 items that yield information that is summarized in five subscales named emotional abuse, physical abuse, sexual abuse, emotional neglect, and physical neglect. For the current purpose a sumscore combining the above scales was computed. Internal consistency for this scale in our study was Cronbach's α = .93.

### 2.4 Statistical analyses

In order to test the relations between age of onset and participant's course and other clinical characteristics we used regression models with age of onset as the independent variable fitting logistic regression models for discrete binary data, ordinal regression models for discrete non-binary ordinal data, and linear regression models for continuous outcome data.

Following previous research we assumed that groups would differ by current age and gender distribution. In order to adjust for potential relationships between age of onset, gender, the interaction of age and gender, and other demographic variables that showed significant differences between groups, we followed up findings from univariate analyses by conducting multivariate analyses in which these variables were forced into the regression model in the first step. Furthermore, in order to explore the potential for mediating effects of length of depression, i.e. time from onset to current age, and early adversity, we ran the above multivariate regression analyses with depression and early adversity force entered alongside the group variable in the second step of the analysis. In order to test whether age of onset was related to the probability of transitioning from suicidal ideation to suicide attempt we examined the proportion of those who reported suicidal ideation (BSS screening) who also reported that they had attempted suicide (BSS item 20) in the pre-adult and adult onset groups. In order to test whether differences in the proportion of those with previous suicide attempts were due to differences in length of the disorder or differences in early adversity we re-ran regression analyses predicting self-reported and interview-derived history of suicide attempt adjusting for both of these factors by force entering them into the equation in a first step before entering the age-of-onset group variable. Finally, generalizability of findings was tested by conducting a sensitivity analysis in which regression analyses were re-run with a cut-off score of 21 years.

## 3 Results

**Fig. 1:**
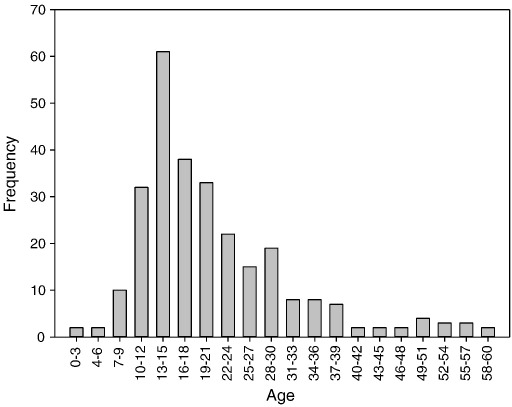
Frequency distribution of age of onset of first episode of major depression.

Age of onset information was available for 275 of the 276 participants recruited into the SWAD-trial. Mean age of onset was 20.91 years (*SD* = 10.81, range: 3–60). Forty-eight percent of the sample (*n* = 132) reported pre-adult onset (onset < 18), 52% (*n* = 143) adult onset (onset ≥ 18). The modal age of onset (13–15 years) replicates the pattern shown by Zisook et al. (2004) in the STAR*D trial (see Fig. 1).

Mean current age in pre-adult onset group was significantly younger than in the adult onset group. The groups also differed in terms of employment status with a higher proportion of participants unemployed in the pre-adult onset group and a higher proportion retired in the adult onset group. There were no significant differences with regard to gender distribution, years of full-time education, or ethnicity (see Table 1).

The proportion of those who had attempted suicide was significantly higher in the group with pre-adult onset than in the group with adult onset, with results consistent for self-reported (binary scored BSS Item 20) and interviewer-rated suicide attempts (SASII). These results remained significant after adjusting for the effect of age, gender, the interaction of age and gender and employment status. Participants with pre-adult onset also reported higher levels of suicidality during their worst episode of depression (SCID). However, these differences were no longer significant when we adjusted for the effect of sociodemographic variables. Analysis of transitional probabilities from suicidal ideation to suicide attempt showed that, of the 87 participants who reported having suffered suicidal ideation in the pre-adult onset group, 42 (48%) had attempted suicide; while of the 65 participants who reported suicidal ideation in the adult onset group only 17 (26%) had attempted, *χ*^2^ = 7.66, *df* = 1, *p* = .006.

Pre-adult onset was associated with a significantly longer duration of depression and a significantly higher number of previous episodes even when adjusted for differences in sociodemographic variables. However, differences in numbers of previous episodes were no longer significant when adjusted for differences in the length of the disorder, *β* = .12, *t* = 1.34, *p* = .18. There were significant differences in levels of early adversity with pre-adult onset being associated with significantly higher levels of early adversity even after adjusting for differences in sociodemographic variables.

There were no significant differences between the two groups in current levels of depressive symptoms and hopelessness. There were also no differences in rates of those with current or past anxiety disorder or current or past substance abuse or dependence. The two groups were comparable with regard to current use of antidepressant medication (see Table 1).

When regression analyses predicting self-reported and interview-derived history of suicide attempt were re-run adjusting for length of the disorder and differences in early adversity, age of onset remained a significant predictor, *Exp* (*B*) = 2.71, Wald = 10.42, *p* = .001, as did early adversity as assessed by CTQ, *Exp* (*B*) = 1.02, Wald = 8.34, *p* = .004, while the contribution of length of the disorder was not significant, *Exp* (*B*) = 1.01, Wald = 1.11, *p* = .292.

When analyses were re-run with a cut-off age of 21, results remained virtually unchanged with the only difference occurring being a significant effect for gender distribution in the analyses using a cut-off age of 21 but no significant effect in the analyses using a cut-off age of 18.

## 4 Discussion

The importance of understanding the effect of pre-adult onset depression cannot be overstated. Pre-adult onset is common and in clinical studies has consistently been linked to increased risk of suicidality and suicide attempts (Benazzi, 2000; Byford et al., 2003; Van Noorden et al., 2011; Zisook et al., 2007). In the current sample, 48% of participants reported an onset before the age of 18, which is similar to rates from previous studies that have recruited for clinical trials (about 35 to 40%, Zisook et al., 2007; Fava et al., 1996; Alpert et al., 1999), but differs significantly from the rate of 22% found in a recent study using a more naturalistic sample (Van Noorden et al., 2011).

The current study allowed us to ascertain whether the pattern of age of onset and suicidality – reported in previous studies of currently depressed patients – extends to clinical samples who are not currently depressed. The results are clear: although all of the participants in our study were currently in remission we found the same pattern of results. First, we confirmed the pattern of age of onset, with the most common period for first episode of MDD being between 13 and 15 years of age. Second, those who reported a pre-adult onset of the disorder were significantly more likely to have attempted suicide in the past than those who reported adult onset of the disorder. Consistent with previous research (Van Noorden et al., 2011; Zisook et al., 2007), these findings remained significant when adjusted for differences in sociodemographic variables such as age, gender, and employment status. Taking into account differences in these variables, participants with early or pre-adult onset showed 3.08 higher odds of having attempted suicide when analyses were based on self-report; 2.19 higher odds when analyses were based on interviewer ratings. Participants with pre-adult onset also reported higher levels of suicidality during their worst episode of depression. However, these differences were less robust and did not remain significant when they were adjusted for the influence of sociodemographic variables reflecting the fact that worst episodes of depression may not necessarily coincide with the episodes in which individuals are most suicidal. Furthermore, we found that in the pre-adult onset group a significantly greater proportion of those who had been suicidal had actually acted on their ideation and attempted suicide. This increase in the transitional probability from ideation to behavior is consistent with previous research showing that individuals with pre-adult onset depression report higher suicidal intent (Thompson, 2008). The results of sensitivity analyses showed that these findings were not restricted to age-of-onset groups defined by a cut-off age of 18 but generalized at least to a cut-off age of 21.

What might explain the relation between age of onset and increased rates of suicidality? Van Noorden et al. (2011) discussed three potential explanations, which our results speak to at least in part. First, pre-adult onset and increased suicidality could index a particular variant of Major Depression that might be characterized by different genetic and familial load or other childhood experiences. While our data did not include any information on familiality and genetic factors, we did find evidence for differences in early adversity. However, further analyses showed that these differences could not explain the relation between pre-adult onset and increased suicidality suggesting that, if such a variant existed, it would not be sufficiently characterized by increased early adversity alone. Second, pre-adult onset could merely index a more severe course of the disorder with higher suicidality simply arising as a consequence of higher overall levels of symptoms. This explanation is also not supported by our findings. Although patients with earlier onset did report higher numbers of previous episodes, this was entirely due to differences in length of the disorder. The groups did not differ with regard to rates of those who had experienced a chronic episode. There were also no differences with regard to previous anxiety or substance-related disorders. A third explanation put forward for the association between pre-adult onset and later morbidity is that increased suicidality might arise as a consequence of the increased length of the disorder. When we adjusted for differences in length of the disorder, the relation between pre-adult onset and increased suicidality remained significant thus suggesting that the relation could not be explained by differences in sheer length of the disorder alone. Of course, this does not rule out the effect of other factors associated with a longer course, which were not assessed here. For example, in an analysis of data from the National Comorbidity Survey Replication, Angst et al. (2010) found that patients with unipolar depression who had clinically significant subthreshold levels of manic symptoms had both earlier onset and higher rates of suicide attempts. While the relation between pre-adult onset and increased suicidality can be considered a robust finding, observations concerning explanations of this effect are clearly still preliminary and more theoretically guided research is now needed to elucidate the biological and psychological mechanisms through which this relation is carried.

The current study has a number of limitations. As in previous studies, relevant variables were all assessed through retrospective reports. Such reports, even if assessed using multiple methods, are prone to reporting error and bias and are generally less reliable than medical records and prospective assessments. Furthermore, the current sample was recruited for the purpose of participation in a clinical trial of treatments for the prevention of relapse to depression. As participants responded to an offer of treatment it is possible that the sample is biased towards more severe presentations. This might have affected the overall rates of pre-adult onset/suicidality observed, but would not be expected to account for the different relationships between these variables such as the increased transitional probability between suicide ideation and suicidal behavior. Finally, our analyses focussed on differences in history of suicidality and while we also assessed a range of variables indicating severity of psychopathology and course of the disorder there are a number of other potentially relevant factors in this context that remained unassessed in our research.

Altogether, our findings replicate, in a sample of patients who are currently in remission, the finding that pre-adult age of onset is closely associated with later suicidality. These findings further stress the importance of taking into account pre-adult age of onset in the assessment of suicide risk, and stress the need for developing and providing early interventions (Korczak and Goldstein, 2009).

## Role of funding source

This research was funded by a Wellcome Trust Programme Grant (067797/Z/02/A) to Prof. JMG Williams and Prof. IT Russell.

## Conflict of interest

None of the authors has any financial involvement or affiliation with any organization whose financial interests may be affected by material in the manuscript, or which might potentially bias it.

## Figures and Tables

**Table 1:** Descriptives and test statistics for the comparison between pre-adult (< 18) and adult (> = 18) onset groups.

	Onset classes		Analysis			
Characteristic	Pre-adult onset (*n* = 132; 48%)	Adult onset (*n* = 143; 52%)	Test statistic	df	p	
Age in years, *M* (*SD*)	39.3 (11.2)	47.0 (11.4)	*F* = 31.48	1	.000	
Gender, *n* female (%)	101 (76)	99 (69)	*χ*^2^ = 1.83	1	.175	
Relationship status			*χ*^2^ = .90	1	.341	
Currently in relationship, *n* (%)	88 (70)	75 (65)				
Currently not in relationship, *n* (%)	37 (30)	41 (35)				
Years of full-time education, *M* (*SD*)	15.2 (3.7)	15.1 (3.0)	*F* = .04	1	.836	
Employment status			*χ*^2^ = 11.70	2	.003	
Employed, *n* (%)	84 (67)	99 (72)				
Unemployed, *n* (%)	35 (28)	19 (14)				
Retired, *n* (%)	7 (5)	20 (14)				
Ethnicity			*χ*^a^ = .19	1	.663	
Caucasian-white, *n* (%)	126 (96)	136 (95)				
Other, *n* (%)	5 (4)	7 (5)				

			Test statistic	df	Unadjusted p	Adjusted p^b^
Suicidality						
Self-reported presence of suicide attempt, *n* (%)^c^ Adjusted odds ratio [95% CI]	50 (39) 3.18 [2.56, 3.80]	23 (16) 1	*χ*^2^ = 16.64	1	.000	.000
Interviewer-rated presence of suicide attempt, *n* (%)^d^ Adjusted odds ratio [95% CI]	66 (50) 2.19 [1.65, 2.79]	38 (26) 1	*χ*^2^ = 16.01	1	.000	.005
Interviewer-rated suicidality during worst episode of depression, *M* (*SD*)^a^	3.00 (1.59)	2.52 (1.63)	*F* = 6.13	1	.014	.103
Course of the disorder						
Length of disorder in years, *M* (*SD*)	26.35 (12.19)	18.83 (12.17)	*F* = 26.12	1	.000	.000
Number of previous episodes, *Mdn* (range)^e^	6 (3–45)	5 (3–18)	*F* = 7.74	1	.006	.001
Presence of chronic episode, *n* (%)^e^	24 (17)	23 (17)	*χ*^2^ = .01	1	.925	.390
Early adversity						
Childhood trauma questionnaire, *M* (*SD*)	46.48 (19.09)	41.16 (15.21)	*F* = 6.51	1	.011	.009
Current symptoms						
Hamilton rating scale for depression, *M* (*SD*)	2.78 (3.13)	3.60 (3.87)	*F* = 3.61	1	.058	.083
Beck depression inventory, *M* (*SD*)	7.34 (7.37)	9.03 (8.54)	*F* = 3.07	1	.081	.375
Beck hopelessness scale, *M* (*SD*)	5.00 (4.70)	5.41 (4.58)	*F* = .50	1	.478	.864
Other emotional disorders						
Current anxiety disorder, *n* (%) e	22 (17)	26 (18)	χ^a^ = .10	1	.741	.599
History of anxiety disorder, *n* (%) e	43 (33)	44 (31)	*χ*^2^ = .10	1	.748	.221
History of substance dependence or Abuse, *n* (%) e	26 (20)	18 (13)	*χ*^2^ = 2.58	1	.108	.378
Medication use						
Currently taking antidepressants, *n* (%) e	51 (44)	70 (54)	*χ*^2^ = 2.59	1	.107	.181

a Assessed through SCID interview rating of level of suicidality during worst episode depression.

b Adjusted for age, gender, age by gender interaction, and employment status.

c Assessed through worst-ever version of the Beck Scale for Suicidal Ideation (BSS) item 20 coded as binary score.

d Assessed through SASSI interview.

e Assessed through SCID interview.
